# Dehydro­brachylaenolide: an eudesmane-type sesquiterpene lactone

**DOI:** 10.1107/S1600536808042402

**Published:** 2008-12-24

**Authors:** M. Rademeyer, F. R. van Heerden, M. M. van der Merwe

**Affiliations:** a Department of Chemistry, University of Pretoria, Pretoria 0002, South Africa; b School of Chemistry, University of KwaZulu-Natal, Pietermaritzburg Campus, Private Bag X01, Scotsville 3209, South Africa; c Biosciences, CSIR, Pretoria, South Africa, and, School of Chemistry, University of KwaZulu-Natal, Pietermaritzburg Campus, Private Bag X01, Scotsville 3209, South Africa

## Abstract

The three-ring eudesmanolide, C_15_H_16_O_3_, is a natural product isolated from *Dicoma anomala* Sond. (Asteraceae). The compound contains an *endo*–*exo* cross conjugated methyl­enecyclo­hexenone ring with an envelope conformation *trans*-fused with cyclo­hexane and *trans*-annelated with an α-methyl­ene γ-lactone. The absolute structure was assigned by optical rotation measurements compared to those from the synthetic compound with known stereochemistry. The crystal packing is consolidated by C—H⋯O interactions.

## Related literature

For NMR studies of this compound, see: Bohlmann & Zdero, (1982 ▶); Grass *et al.* (2004 ▶). For the chemical synthesis and confirmation of the absolute structure, see: Higuchi *et al.* (2003 ▶).
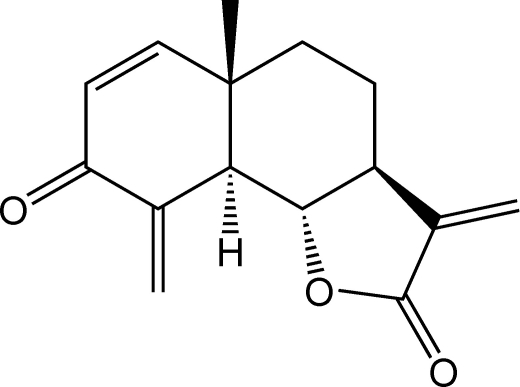


## Experimental

### 

#### Crystal data

C_15_H_16_O_3_
*M* *_r_* = 244.28
Orthorhombic, 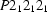

*a* = 9.5648 (6) Å
*b* = 11.1631 (6) Å
*c* = 11.5542 (6) Å
*V* = 1233.67 (12) Å^3^
*Z* = 4
Mo *K*α radiation
μ = 0.09 mm^−1^
*T* = 150 (2) K
0.50 × 0.50 × 0.40 mm

#### Data collection

Oxford Diffraction Excalibur2 CCD diffractometer
Absorption correction: multi-scan (Blessing, 1995 ▶) *T*_min_ = 0.909, *T*_max_ = 0.963
12604 measured reflections
2294 independent reflections
1988 reflections with *I* > 2σ(*I*)
*R*_int_ = 0.016

#### Refinement

*R*[*F* ^2^ > 2σ(*F* ^2^)] = 0.033
*wR*(*F* ^2^) = 0.095
*S* = 1.05
2294 reflections
163 parameters
H-atom parameters constrained
Δρ_max_ = 0.31 e Å^−3^
Δρ_min_ = −0.21 e Å^−3^

### 

Data collection: *CrysAlis CCD* (Oxford Diffraction, 2006 ▶); cell refinement: *CrysAlis RED* (Oxford Diffraction, 2006 ▶); data reduction: *CrysAlis RED*; program(s) used to solve structure: *SHELXS97* (Sheldrick, 2008 ▶); program(s) used to refine structure: *SHELXL97* (Sheldrick, 2008 ▶); molecular graphics: *Mercury* (Macrae *et al.*, 2006 ▶); software used to prepare material for publication: *PLATON* (Spek, 2003 ▶) and *WinGX* (Farrugia, 1999 ▶).

## Supplementary Material

Crystal structure: contains datablocks global, I. DOI: 10.1107/S1600536808042402/bi2330sup1.cif

Structure factors: contains datablocks I. DOI: 10.1107/S1600536808042402/bi2330Isup2.hkl

Additional supplementary materials:  crystallographic information; 3D view; checkCIF report

## Figures and Tables

**Table 1:** Hydrogen-bond geometry (Å, °)

*D*—H⋯*A*	*D*—H	H⋯*A*	*D*⋯*A*	*D*—H⋯*A*
C6—H6⋯O1^i^	0.98	2.39	3.360 (2)	171
C14—H14*A*⋯O1^i^	0.96	2.57	3.393 (2)	143

Symmetry code: (i) 
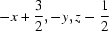
.

**Fig. 1.**
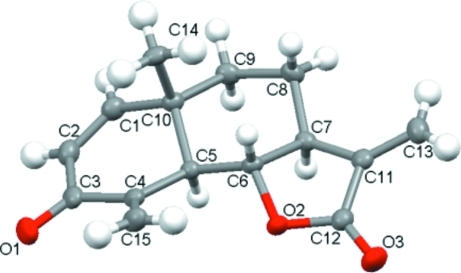
Molecular structure showing displacement ellipsoids at 50% probability for all atoms.

## supplementary crystallographic information

### Comment

The title compound, a sesquiterpene lactone dehydrobrachylaenolide, was isolated from *Dicoma anomala* Sond (Asteraceae). These bi-functional *exo*-*endo* cross conjugated dienones are of importance as synthetic intermediates in the preparation of biologically active natural products (Higuchi *et al.*, 2003). NMR studies of the compound have been reported previously (Bohlmann & Zdero, 1982; Grass *et al.*, 2004) and the absolute stereochemistry has been confirmed as 3-oxoeudesma-1,4(15),11 (13)-triene-12,6a-olide by chemical synthesis (Higuchi *et al.*, 2003). Here we report the crystal structure. Although the absolute structure could not be elucidated by X-ray diffraction, unambiguous assignment of stereochemistry was made on the basis of the value of optical rotation ([α] ^24^_D_+68° (c 1/2, CHCl_3_)) which is identical to that of the synthetic compound ([α] ^24^_D_+67.9° (c 0.16, CHCl_3_)) for which the stereochemistry is known (Higuchi *et al.*, 2003) and very close to the value for the naturally isolated material ([α]^24^_D_+67° (c 0.16, CHCl_3_)) (Bohlmann & Zdero, 1982).

The molecular geometry and labelling scheme are shown in Fig. 1. The methylenecyclohexenone ring adopts an envelope conformation, with the C5 atom out of the plane of the ring by approximately 0.7 Å. The γ-lactone ring is twisted on C6—C7, while the cyclohexane ring adopts a chair conformation. An axial position is occupied by methyl group C14, and the methylene carbon atom C15 is in the equatorial position. A weak intramolecular interaction is formed between C15—H15B···O2. Fig. 2 illustrates the molecular packing viewed down the *c* axis. Weak intermolecular hydrogen bonds are present between atoms C6—H6···O1^i^ and and C14—H14···O1^i^ [symmetry code (i): 1/2 - *x*, 1 - *y*, 1/2 + *z*].

### Experimental

The compound was isolated from *Dicoma anomala* Sond (Asteraceae), and recrystallized from propanol at room temperature.

### Refinement

H atoms were placed geometrically and refined in idealized positions in the riding-model approximation, with C—H = 0.93–0.98 Å with *U*_iso_(H) = 1.2 or 1.5*U*_eq_(C). In the absence of significant anomalous scattering effects, Friedel pairs were merged as equivalent data.

### Crystal data

**Table:**

C_15_H_16_O_3_	*F*(000) = 520
*M**_r_* = 244.28	*D*_x_ = 1.315 Mg m^−^^3^
Orthorhombic, *P*2_1_2_1_2_1_	Mo *K*α radiation, λ = 0.71073 Å
Hall symbol: P 2ac 2ab	Cell parameters from 8167 reflections
*a* = 9.5648 (6) Å	θ = 4.0–31.8°
*b* = 11.1631 (6) Å	µ = 0.09 mm^−^^1^
*c* = 11.5542 (6) Å	*T* = 150 K
*V* = 1233.67 (12) Å^3^	Block, colourless
*Z* = 4	0.50 × 0.50 × 0.40 mm

### Data collection

**Table:**

Oxford Diffraction Excalibur2 CCD diffractometer	2294 independent reflections
Radiation source: fine-focus sealed tube	1988 reflections with *I* > 2σ(*I*)
graphite	*R*_int_ = 0.016
ω scans	θ_max_ = 31.9°, θ_min_ = 4.0°
Absorption correction: multi-scan (Blessing, 1995)	*h* = −13→13
*T*_min_ = 0.909, *T*_max_ = 0.963	*k* = −15→16
12604 measured reflections	*l* = −16→17

### Refinement

**Table:**

Refinement on *F*^2^	Primary atom site location: structure-invariant direct methods
Least-squares matrix: full	Secondary atom site location: difference Fourier map
*R*[*F*^2^ > 2σ(*F*^2^)] = 0.033	Hydrogen site location: inferred from neighbouring sites
*wR*(*F*^2^) = 0.095	H-atom parameters constrained
*S* = 1.05	*w* = 1/[σ^2^(*F*_o_^2^) + (0.0708*P*)^2^] where *P* = (*F*_o_^2^ + 2*F*_c_^2^)/3
2294 reflections	(Δ/σ)_max_ = 0.001
163 parameters	Δρ_max_ = 0.31 e Å^−^^3^
0 restraints	Δρ_min_ = −0.21 e Å^−^^3^

### Special details

**Table:**

Geometry. All e.s.d.'s (except the e.s.d. in the dihedral angle between two l.s. planes) are estimated using the full covariance matrix. The cell e.s.d.'s are taken into account individually in the estimation of e.s.d.'s in distances, angles and torsion angles; correlations between e.s.d.'s in cell parameters are only used when they are defined by crystal symmetry. An approximate (isotropic) treatment of cell e.s.d.'s is used for estimating e.s.d.'s involving l.s. planes.
Refinement. Refinement of *F*^2^ against ALL reflections. The weighted *R*-factor *wR* and goodness of fit *S* are based on *F*^2^, conventional *R*-factors *R* are based on *F*, with *F* set to zero for negative *F*^2^. The threshold expression of *F*^2^ > σ(*F*^2^) is used only for calculating *R*-factors(gt) *etc*. and is not relevant to the choice of reflections for refinement. *R*-factors based on *F*^2^ are statistically about twice as large as those based on *F*, and *R*- factors based on ALL data will be even larger.

### Fractional atomic coordinates and isotropic or equivalent isotropic displacement parameters (Å^2^)

**Table:**

	*x*	*y*	*z*	*U*_iso_*/*U*_eq_	
C4	0.86180 (14)	0.08184 (11)	1.24094 (11)	0.0237 (2)	
O2	0.87541 (10)	0.20733 (7)	1.00141 (7)	0.0245 (2)	
C7	1.04877 (12)	0.07144 (11)	0.94161 (10)	0.0210 (2)	
H7	1.1253	0.1211	0.9714	0.025*	
C8	1.10043 (14)	−0.05782 (12)	0.94001 (11)	0.0246 (3)	
H8A	1.1798	−0.0657	0.8883	0.029*	
H8B	1.0268	−0.1109	0.9136	0.029*	
C9	1.14347 (13)	−0.08979 (11)	1.06503 (11)	0.0245 (2)	
H9A	1.1717	−0.1732	1.0675	0.029*	
H9B	1.2238	−0.0416	1.0866	0.029*	
O3	0.85244 (12)	0.31953 (9)	0.84156 (9)	0.0372 (3)	
C14	0.90759 (14)	−0.16252 (11)	1.13671 (12)	0.0270 (3)	
H14A	0.8694	−0.1540	1.0603	0.041*	
H14B	0.8354	−0.1493	1.1930	0.041*	
H14C	0.9447	−0.2419	1.1458	0.041*	
C6	0.92565 (13)	0.08521 (10)	1.02492 (10)	0.0197 (2)	
H6	0.8525	0.0278	1.0037	0.024*	
C3	0.90807 (15)	0.04878 (12)	1.36063 (11)	0.0274 (3)	
O1	0.84841 (13)	0.08529 (10)	1.44767 (9)	0.0385 (3)	
C11	0.98993 (14)	0.13415 (11)	0.83767 (11)	0.0234 (2)	
C1	1.08111 (15)	−0.08814 (12)	1.27617 (12)	0.0279 (3)	
H1	1.1553	−0.1409	1.2867	0.033*	
C5	0.97025 (12)	0.06224 (10)	1.14800 (10)	0.0197 (2)	
H5	1.0491	0.1156	1.1647	0.024*	
C10	1.02589 (13)	−0.06954 (10)	1.15444 (10)	0.0213 (2)	
C15	0.73174 (15)	0.12080 (12)	1.22442 (14)	0.0321 (3)	
H15A	0.6708	0.1272	1.2868	0.039*	
H15B	0.7019	0.1416	1.1505	0.039*	
C13	1.00061 (16)	0.11111 (13)	0.72528 (11)	0.0303 (3)	
H13A	0.9501	0.1564	0.6722	0.036*	
H13B	1.0586	0.0497	0.6996	0.036*	
C12	0.89936 (14)	0.23105 (11)	0.88715 (11)	0.0263 (3)	
C2	1.02875 (16)	−0.03277 (13)	1.36888 (11)	0.0304 (3)	
H2	1.0696	−0.0460	1.4408	0.036*	

### Atomic displacement parameters (Å^2^)

**Table:**

	*U*^11^	*U*^22^	*U*^33^	*U*^12^	*U*^13^	*U*^23^
C4	0.0291 (6)	0.0164 (5)	0.0257 (5)	−0.0021 (5)	0.0069 (5)	0.0005 (4)
O2	0.0309 (5)	0.0174 (4)	0.0253 (4)	0.0031 (3)	0.0029 (4)	0.0023 (3)
C7	0.0202 (5)	0.0206 (5)	0.0221 (5)	−0.0010 (4)	0.0013 (4)	−0.0030 (4)
C8	0.0239 (6)	0.0254 (6)	0.0244 (5)	0.0037 (5)	0.0003 (5)	−0.0048 (5)
C9	0.0200 (5)	0.0245 (6)	0.0289 (6)	0.0036 (5)	−0.0014 (5)	−0.0027 (4)
O3	0.0499 (7)	0.0262 (5)	0.0356 (5)	0.0061 (5)	0.0000 (5)	0.0081 (4)
C14	0.0277 (6)	0.0168 (5)	0.0366 (6)	−0.0020 (5)	−0.0027 (5)	0.0014 (5)
C6	0.0195 (5)	0.0148 (5)	0.0248 (5)	0.0006 (4)	0.0012 (4)	−0.0001 (4)
C3	0.0330 (6)	0.0233 (6)	0.0259 (5)	−0.0079 (5)	0.0076 (5)	0.0017 (5)
O1	0.0493 (6)	0.0366 (6)	0.0297 (5)	−0.0062 (5)	0.0165 (5)	−0.0008 (4)
C11	0.0233 (6)	0.0207 (5)	0.0261 (5)	−0.0040 (4)	0.0015 (5)	0.0001 (4)
C1	0.0288 (6)	0.0252 (6)	0.0297 (6)	0.0015 (5)	−0.0043 (5)	0.0036 (5)
C5	0.0202 (5)	0.0171 (5)	0.0217 (5)	−0.0007 (4)	0.0021 (4)	−0.0002 (4)
C10	0.0212 (5)	0.0186 (5)	0.0241 (5)	0.0011 (4)	−0.0022 (4)	0.0000 (4)
C15	0.0310 (7)	0.0263 (6)	0.0391 (7)	0.0033 (5)	0.0129 (6)	0.0040 (6)
C13	0.0314 (6)	0.0337 (7)	0.0257 (6)	−0.0050 (6)	0.0028 (6)	0.0006 (5)
C12	0.0303 (6)	0.0219 (6)	0.0266 (6)	−0.0028 (5)	−0.0002 (5)	0.0019 (4)
C2	0.0358 (7)	0.0301 (6)	0.0253 (6)	−0.0048 (5)	−0.0011 (5)	0.0048 (5)

### Geometric parameters (Å, °)

**Table:**

C4—C15	1.332 (2)	C14—H14B	0.960
C4—C3	1.4982 (18)	C14—H14C	0.960
C4—C5	1.5090 (16)	C6—C5	1.5067 (16)
O2—C12	1.3659 (15)	C6—H6	0.980
O2—C6	1.4708 (14)	C3—O1	1.2260 (16)
C7—C11	1.4997 (17)	C3—C2	1.473 (2)
C7—C8	1.5253 (18)	C11—C13	1.3278 (18)
C7—C6	1.5287 (16)	C11—C12	1.4991 (18)
C7—H7	0.980	C1—C2	1.334 (2)
C8—C9	1.5439 (18)	C1—C10	1.5167 (17)
C8—H8A	0.970	C1—H1	0.930
C8—H8B	0.970	C5—C10	1.5662 (15)
C9—C10	1.5437 (17)	C5—H5	0.980
C9—H9A	0.970	C15—H15A	0.930
C9—H9B	0.970	C15—H15B	0.930
O3—C12	1.2059 (16)	C13—H13A	0.930
C14—C10	1.5490 (17)	C13—H13B	0.930
C14—H14A	0.960	C2—H2	0.930
			
C15—C4—C3	119.26 (12)	O1—C3—C2	121.14 (13)
C15—C4—C5	125.99 (12)	O1—C3—C4	122.53 (13)
C3—C4—C5	114.71 (11)	C2—C3—C4	116.32 (11)
C12—O2—C6	107.66 (9)	C13—C11—C12	123.86 (13)
C11—C7—C8	123.60 (10)	C13—C11—C7	131.60 (13)
C11—C7—C6	99.69 (10)	C12—C11—C7	104.38 (10)
C8—C7—C6	110.63 (10)	C2—C1—C10	123.39 (12)
C11—C7—H7	107.3	C2—C1—H1	118.3
C8—C7—H7	107.3	C10—C1—H1	118.3
C6—C7—H7	107.3	C6—C5—C4	116.89 (10)
C7—C8—C9	107.08 (10)	C6—C5—C10	107.51 (9)
C7—C8—H8A	110.3	C4—C5—C10	109.63 (9)
C9—C8—H8A	110.3	C6—C5—H5	107.5
C7—C8—H8B	110.3	C4—C5—H5	107.5
C9—C8—H8B	110.3	C10—C5—H5	107.5
H8A—C8—H8B	108.6	C1—C10—C9	110.29 (10)
C10—C9—C8	113.46 (10)	C1—C10—C14	106.58 (10)
C10—C9—H9A	108.9	C9—C10—C14	110.22 (10)
C8—C9—H9A	108.9	C1—C10—C5	106.91 (10)
C10—C9—H9B	108.9	C9—C10—C5	110.69 (9)
C8—C9—H9B	108.9	C14—C10—C5	112.02 (9)
H9A—C9—H9B	107.7	C4—C15—H15A	120.0
C10—C14—H14A	109.5	C4—C15—H15B	120.0
C10—C14—H14B	109.5	H15A—C15—H15B	120.0
H14A—C14—H14B	109.5	C11—C13—H13A	120.0
C10—C14—H14C	109.5	C11—C13—H13B	120.0
H14A—C14—H14C	109.5	H13A—C13—H13B	120.0
H14B—C14—H14C	109.5	O3—C12—O2	121.23 (12)
O2—C6—C5	115.13 (9)	O3—C12—C11	129.75 (13)
O2—C6—C7	103.21 (9)	O2—C12—C11	109.00 (10)
C5—C6—C7	111.05 (10)	C1—C2—C3	121.90 (12)
O2—C6—H6	109.1	C1—C2—H2	119.1
C5—C6—H6	109.1	C3—C2—H2	119.1
C7—C6—H6	109.1		
			
C11—C7—C8—C9	−176.53 (11)	C15—C4—C5—C10	−124.67 (13)
C6—C7—C8—C9	−58.75 (13)	C3—C4—C5—C10	52.90 (13)
C7—C8—C9—C10	55.27 (14)	C2—C1—C10—C9	151.41 (13)
C12—O2—C6—C5	153.05 (11)	C2—C1—C10—C14	−88.95 (15)
C12—O2—C6—C7	31.88 (12)	C2—C1—C10—C5	31.01 (17)
C11—C7—C6—O2	−39.39 (11)	C8—C9—C10—C1	−173.06 (11)
C8—C7—C6—O2	−171.01 (9)	C8—C9—C10—C14	69.52 (13)
C11—C7—C6—C5	−163.29 (9)	C8—C9—C10—C5	−54.96 (13)
C8—C7—C6—C5	65.10 (12)	C6—C5—C10—C1	175.55 (10)
C15—C4—C3—O1	−20.6 (2)	C4—C5—C10—C1	−56.42 (12)
C5—C4—C3—O1	161.68 (12)	C6—C5—C10—C9	55.39 (12)
C15—C4—C3—C2	158.27 (13)	C4—C5—C10—C9	−176.57 (10)
C5—C4—C3—C2	−19.48 (16)	C6—C5—C10—C14	−68.06 (12)
C8—C7—C11—C13	−19.4 (2)	C4—C5—C10—C14	59.98 (12)
C6—C7—C11—C13	−142.26 (15)	C6—O2—C12—O3	170.70 (12)
C8—C7—C11—C12	155.99 (11)	C6—O2—C12—C11	−10.45 (13)
C6—C7—C11—C12	33.14 (12)	C13—C11—C12—O3	−21.0 (2)
O2—C6—C5—C4	58.69 (14)	C7—C11—C12—O3	163.19 (14)
C7—C6—C5—C4	175.49 (10)	C13—C11—C12—O2	160.33 (12)
O2—C6—C5—C10	−177.59 (9)	C7—C11—C12—O2	−15.53 (13)
C7—C6—C5—C10	−60.79 (12)	C10—C1—C2—C3	2.2 (2)
C15—C4—C5—C6	−2.05 (18)	O1—C3—C2—C1	169.28 (13)
C3—C4—C5—C6	175.53 (10)	C4—C3—C2—C1	−9.6 (2)

### Hydrogen-bond geometry (Å, °)

**Table:**

*D*—H···*A*	*D*—H	H···*A*	*D*···*A*	*D*—H···*A*
C6—H6···O1^i^	0.98	2.39	3.360 (2)	171
C14—H14A···O1^i^	0.96	2.57	3.393 (2)	143

Symmetry codes: (i) −*x*+3/2, −*y*, *z*−1/2.
